# Cognitive and neurodevelopmental outcomes in spinal muscular atrophy: a scoping review

**DOI:** 10.3389/fncel.2026.1842827

**Published:** 2026-06-19

**Authors:** Martina Gnazzo, Giulia Pisanò, Valentina Baldini, Sara Giordani, Laura Caiazza, Benedetta Piccolo, Emanuela Turco, Susanna Esposito, Maria Carmela Pera

**Affiliations:** 1 Department of Mental Health, Physical and Preventive Medicine, Clinic of Child and Adolescent Neuropsychiatry, University of Campania “Luigi Vanvitelli”, Naples, Italy; 2 IRCCS Istituto Delle Scienze Neurologiche di Bologna (ISNB), Bologna, Italy; 3 Dipartimento Materno-Infantile Presidio Ospedaliero Santa Maria Nuova, AUSL-IRCCS Reggio Emilia, Reggio Emilia, Italy; 4 Department of Biomedical and Neuromotor Sciences, University of Bologna, Bologna, Italy; 5 Department of Medicine and Surgery, Pediatric Clinic, University of Parma, Parma, Italy; 6 Department of Biomedical, Metabolic, and Neural Sciences, University of Modena and Reggio Emilia, Modena, Italy; 7 Child Neuropsychiatry Unit, Department of Medicine and Surgery, University of Parma, Italy

**Keywords:** behavioral outcomes, cognition, disease-modifying therapies, executive function, neurodevelopment, spinal muscular atrophy

## Abstract

Spinal muscular atrophy (SMA) has traditionally been described as a motor neuron disorder; however, increasing evidence suggests a broader neurodevelopmental involvement, particularly in the era of disease-modifying therapies. As survival and motor outcomes improve, cognitive and behavioral trajectories have become clinically relevant but remain inconsistently characterized. To systematically synthesize current evidence on cognitive and neurodevelopmental outcomes in children and adults with SMA, with particular focus on differences across phenotypes and therapeutic eras. A scoping review was conducted including observational studies, clinical cohorts, and case series reporting cognitive, language, behavioral, or executive outcomes in individuals with SMA. Studies were analyzed qualitatively with attention to disease severity, age, and treatment status. Twenty-three studies were included. Global intellectual functioning was generally preserved, particularly in SMA types II and III. Nevertheless, selective vulnerabilities were frequently reported in processing speed, executive functioning, and language development. Behavioral and socio-emotional challenges were described in pediatric populations. Neurodevelopmental outcomes in early-onset SMA showed substantial heterogeneity, ranging from global developmental delay to relatively preserved cognitive trajectories, especially in pre-symptomatically treated patients. Methodological variability and motor-related testing limitations were common across studies. Cognitive functioning in SMA appears largely preserved in milder phenotypes but domain-specific vulnerabilities in processing speed, working memory, and language are consistently identified across phenotype groups and represent clinically relevant targets for monitoring and intervention. Systematic neurodevelopmental monitoring and standardized assessment protocols are needed to better define long-term outcomes as treatment modifies disease trajectories.

## Introduction

Spinal muscular atrophy (SMA) is a progressive autosomal recessive neuromuscular disorder caused by biallelic mutations in the *SMN1* gene, resulting in deficiency of the survival motor neuron (SMN) protein, degeneration of *α*-motor neurons, and severe proximal muscle weakness (Mercuri et al., 2022). Although traditionally classified as a lower motor neuron disease, the SMN protein is ubiquitously expressed and performs multiple cellular functions—including snRNP biogenesis, axonal mRNA transport, and local translation at synaptic terminals—that extend its relevance well beyond the motor axis (Detering et al., 2022). In the developing brain, experimental models have demonstrated impaired hippocampal neurogenesis and widespread proteomic dysregulation when SMN levels are reduced (Wishart et al., 2010), and neuroimaging studies have documented structural changes in subcortical regions in severely affected individuals (Lécuyer et al., 2023), collectively suggesting that SMN deficiency may disrupt neurodevelopmental processes during critical periods of brain maturation.

Early clinical observations noted preserved or above-average intellectual functioning in children with SMA types II and III, with some reports documenting enhanced verbal and crystallized intelligence in older children and adolescents—findings tentatively attributed to a compensatory process whereby motor restriction may redirect cognitive resources toward verbal and academic domains (von Gontard et al., 2002; Mix et al., 2021; Lenzoni et al., 2022). Whether this reflects neuroplastic reorganization or environmentally mediated learning strategies remains debated. Systematic cognitive assessment in SMA type I was meanwhile severely constrained by limited survival and profound motor and bulbar impairment, leaving this population largely inaccessible to formal evaluation.

The introduction of disease-modifying therapies—nusinersen, risdiplam, and onasemnogene abeparvovec—has profoundly changed the natural history of SMA (Mercuri et al., 2022; Pera and Mercuri, 2024), enabling survival and motor gains in cohorts previously inaccessible to longitudinal study. As treated children with early-onset SMA reach school age and beyond, attention has shifted toward neurodevelopmental trajectories beyond motor function. Emerging reports have documented cognitive, language, and behavioral difficulties in treated type I patients at rates ranging from 9 to 60%, with particular concerns regarding autism spectrum disorder, language delay, and executive dysfunction (Coratti et al., 2025; Buchignani et al., 2023); these findings have been further contextualized in recent expert commentaries highlighting the emerging spectrum of neurodevelopmental comorbidities in this population (Baranello and Neurodevelopment in SMA Working Group, 2024).

Despite growing clinical interest, the evidence base remains methodologically heterogeneous, with considerable variability in assessment tools, SMA types represented, treatment status, and age ranges examined. Motor-related assessment bias poses a particular challenge, as standard cognitive instruments frequently rely on motor or verbal execution, risking systematic underestimation in patients with significant physical limitations (Giannotta et al., 2024). Broader neurodevelopmental domains—including executive functions, adaptive behavior, and social cognition—have only recently begun to receive systematic attention, and the influence of therapy type and timing on these outcomes remains poorly characterized. A comprehensive and updated synthesis is therefore warranted.

The present study aims to map the current evidence on cognitive and neurodevelopmental outcomes across SMA phenotypes, to identify methodological trends and assessment approaches, and to highlight emerging clinical questions in this evolving field.

## Materials and methods

### Study design

This study was conducted as a scoping review in accordance with the methodological framework proposed by the Joanna Briggs Institute (JBI) (Peters et al., 2020). Reporting was guided by the Preferred Reporting Items for Systematic Reviews and Meta-Analyses extension for Scoping Reviews (PRISMA-ScR) (Tricco et al., 2018). Methodological quality of included studies was independently appraised using the Newcastle-Ottawa Scale (NOS), adapted for cross-sectional studies, which evaluates study quality across the domains of selection, comparability, and outcome assessment. Given the exploratory nature of scoping reviews, appraisal results were considered descriptively and did not serve as grounds for study exclusion.

### Search strategy

A systematic literature search was performed in PubMed/MEDLINE from database inception to February 28, 2026. The search combined terms related to spinal muscular atrophy with terms covering cognitive, neurodevelopmental, and behavioral outcomes, including autism spectrum disorder, language, and executive functions, to reflect the evolving clinical terminology in the post–disease-modifying therapy era. The complete search string is reported.

To mitigate potential retrieval bias, reference lists of all included articles were manually screened to identify additional relevant publications not captured by the electronic search.

Only studies involving human participants and published in English were considered.

### Eligibility criteria

Studies were included if they met all of the following criteria: (1) participants diagnosed with any SMA type (I–IV), regardless of age; (2) quantitative assessment of cognitive and/or neurodevelopmental outcomes using standardized or structured tools; (3) original research articles published in peer-reviewed journals.

Studies were excluded if they: focused exclusively on motor outcomes without any cognitive or neurodevelopmental assessment; relied solely on unstructured caregiver-reported impressions without standardized evaluation; were case reports, editorials, conference abstracts, or narrative reviews; or included SMA only as a control group for another neurological condition.

### Study selection

**Figure 1:**
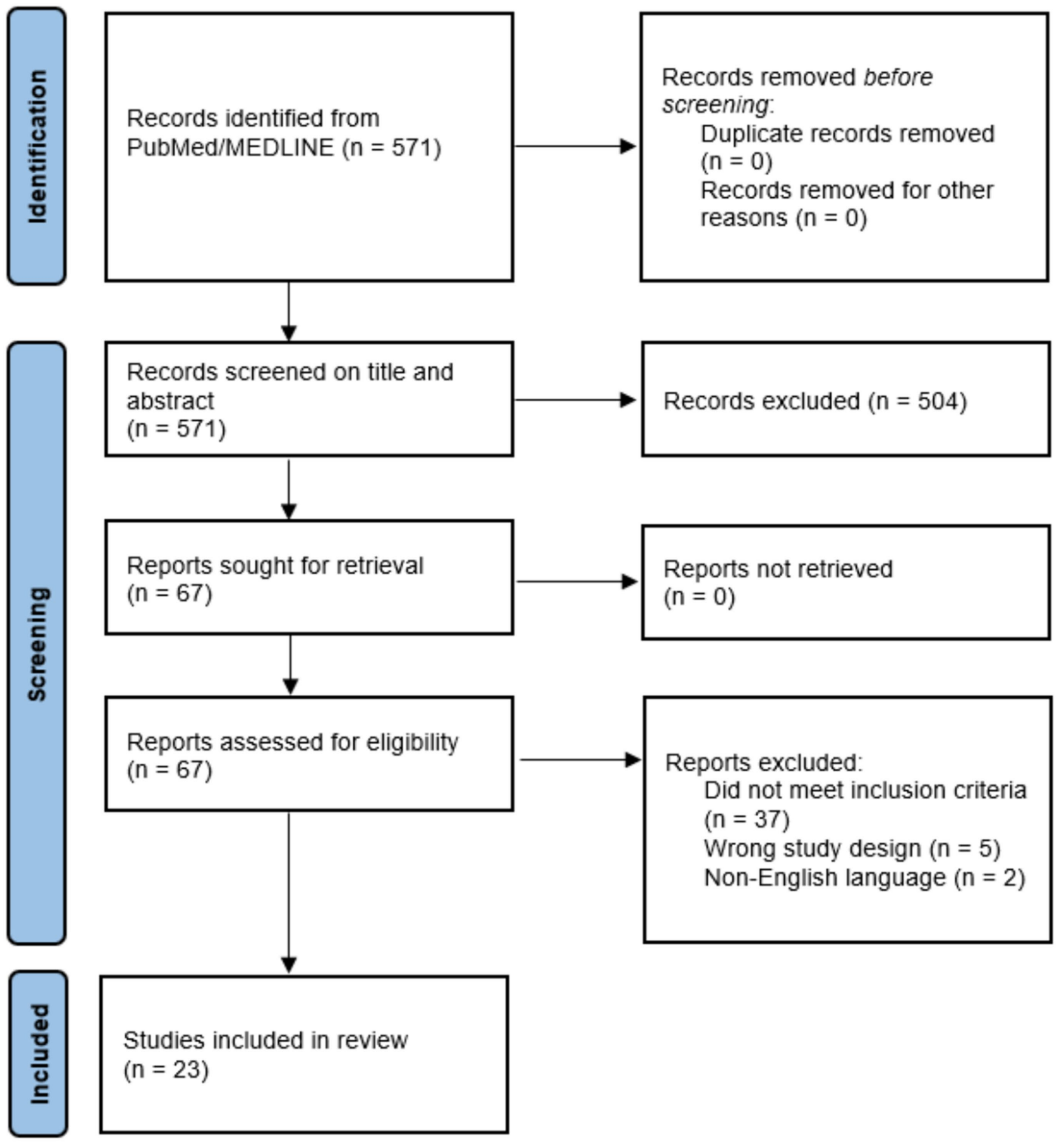
PRISMA 2020 flow diagram of the study selection process. Adapted from: Page et al., 2021.

Records were screened independently by two reviewers (Martina Gnazzo and Sara Giordani) based on titles and abstracts. Full-text articles of potentially eligible studies were then assessed against the inclusion and exclusion criteria. Discrepancies at any stage were resolved through discussion until consensus was reached. The selection process is documented in a PRISMA flow diagram (Figure 1).

### Data extraction and synthesis

Data were extracted independently by two reviewers (Martina Gnazzo and Sara Giordani) using a standardized form and summarized in a comprehensive evidence table. Extracted variables included study design, sample size, SMA type, treatment status, age, cognitive assessment tools, and main findings. Where information was not reported or incompletely described in the original publication, this was recorded as not available and no imputation was performed.

Given the exploratory nature of scoping reviews, results were synthesized descriptively, with attention to emerging domains, assessment approaches, and clinical trends across the full spectrum of SMA phenotypes.

## Results

### Study selection

The PubMed literature search from database inception to February 28, 2026 identified 571 records. After screening of titles and abstracts, 67 full-text articles were assessed for eligibility, of which 23 met the inclusion criteria and were included in the final synthesis. The main reasons for exclusion of the 44 full-text articles were: failure to meet inclusion criteria (*n* = 37), wrong study design (*n* = 5), and non-English language (*n* = 2). A PRISMA flow diagram details the full selection process (Figure 1).

A structured overview of cognitive assessment tools across SMA phenotypes is presented in Table 1, which summarizes instruments used, SMA types assessed, and age ranges covered across included studies. A total of 23 studies (Table 2) were included in the final synthesis, encompassing a wide spectrum of spinal muscular atrophy (SMA) phenotypes, age ranges, and therapeutic statuses. Sample sizes ranged from small observational cohorts to larger multicenter studies. Most recent investigations focused on patients receiving disease-modifying therapies, reflecting the progressive shift toward long-term developmental outcomes in the contemporary treatment era.

**Table 1:** Cognitive profile across SMA phenotypes.

Domain	SMA type	Assessment tools (reference)
Global cognition	SMA I	BSID-III (Kölbel et al., 2024; Steffens et al., 2024); Griffiths III (Bitetti et al., 2024)
	SMA II-III	WPPSI/WISC (Buchignani et al., 2023); WAIS-IV (Kizina et al., 2021); Raven Matrices (Lenzoni et al., 2022)
	Adults SMA II-III	WAIS-IV (Kizina et al., 2021); MoCA (Hu et al., 2023)
Executive functions	SMA II-III	ECAS (Mix et al., 2021); WCST, Stroop, TMT (Lenzoni et al., 2022)
	Adults SMA II-III	ECAS (Vidovic et al., 2023); ANT (Hu et al., 2023)
Language	SMA I	CDI/MB-CDIs (Buchignani et al., 2024; Brusa et al., 2025); TCGB (Zappa et al., 2021)
	SMA II-III	EVALO/EVALEO (Guibert et al., 2026)
Behavioral/socio-emotional	Pediatric SMA I	SDQ (Balaji et al., 2026); SCQ, CARS2 (Buchignani et al., 2025)
Developmental profile	SMA I	Bayley-III (Ngawa et al., 2023; Gandolfi et al., 2025); ASQ-3 (Balaji et al., 2025)

**Table 2:** Summary of studies investigating cognitive functioning in spinal muscular atrophy.

Author, year	Sample size	Mean age (*M* ± SD)	Female sex (%)	Type of SMA	DMT therapy	Cognitive assessment	Cognitive results	Main findings	NOS score
Balaji et al., 2026	48	8.6 ± 3.6	52.1%	Type 1: 21 (43.8%); Type 2: 13 (27.0%); Type 3: 14 (29.2%)	All participants treated with Disease-modifying therapies (distribution not reported)	SDQ; PedsQL-NM	Abnormal SDQ scores in 35.4% of children; difficulties in ≥1 domain in 60.4%; hyperactivity/inattention 25%, emotional problems 25%, conduct problems 25%	Children with SMA showed higher rates of emotional and behavioral difficulties than the general population, associated with lower quality of life scores	5
Balaji et al., 2025	37	Median 43 months, IQR 26.5–53.5	54%	Early-onset SMA (Type 1 phenotype or at-risk based on SMN2 ≤ 3 copies)	All patients received Disease-modifying therapies (nusinersen, risdiplam, or onasemnogene abeparvovec; distribution not fully reported)	ASQ-3; M-CHAT-R	43.2% no/low developmental risk; 21.6% isolated gross motor delay; 8.1% isolated non-motor delay; 27.0% global developmental delay; 87.5% low ASD risk	Heterogeneous neurodevelopmental outcomes; better outcomes associated with higher SMN2 copy number, early diagnosis via newborn screening, clinically silent status at treatment initiation, better motor and bulbar function, and absence of parental mental health conditions, highlighting both biological and psychosocial determinants	7
Bitetti et al., 2024	12	Mean age at baseline 6.8 months (range 2–14)	33.3%	SMA type 1 (all with 2 SMN2 copies)	Onasemnogene abeparvovec; 9 previously treated with nusinersen	Griffiths III scales of child development	Significant increases at 12 months in Foundations of Learning, Language and Communication, Eye-Hand Coordination, and Personal-Social–Emotional subscales; no improvement in Gross Motor subscale	Gene therapy led to improvement in motor function (CHOP-INTEND), motor milestones, and language/cognitive development, although many children still showed overall developmental delay after 12 months	7
Buchignani et al., 2025	31	5.70 ± 2.0 years	50%	SMA type I and presymptomatic SMA identified by newborn screening	All patients received at least one disease-modifying therapy (nusinersen, risdiplam, or onasemnogene abeparvovec; distribution not fully reported)	Wechsler scales, Leiter, Raven matrices, developmental scales; behavioral assessment with SDQ, SCQ, SP2; neurobehavioral observation; CARS2; DSM-5 criteria	51% normal cognitive level, 19% borderline, 29% intellectual disability; ASD diagnosed in 3/31 (~10%); additional disorders in 5/31 (ODD, anxiety, ADHD); 14/31 showed abnormal prosocial SDQ scores, 6/31 borderline SCQ	Cognitive profile is heterogeneous with a substantial proportion of intellectual disability; ASD prevalence slightly higher than general population; other neurobehavioral disorders less frequent; assessment is complicated by motor impairment affecting validity of standard tools; need for tailored diagnostic approaches combining screening, observation, and clinical evaluation	6
Buchignani et al., 2023	57	9.15 ± 3.26 years	50.9%	SMA type II (66.7%) and III (33.3%)	95% treated (94% nusinersen, 6% risdiplam)	WPPSI-III (preschool) and WISC-IV (school age)	72% normal IQ, 11% above average, 14% borderline, 3% mild intellectual disability. Lower scores in working memory and processing speed	Intellectual disability is rare in SMA II-III; cognitive profile largely normal but processing speed correlates with motor function, suggesting motor impairment may affect performance in some cognitive tasks	7
Buchignani et al., 2024	24	Mean age 22 months (range 8–42)	54%	SMA type 1 symptomatic (*n* = 14) and presymptomatic infants identified by screening (*n* = 10)	Nusinersen or Onasemnogene abeparvovec (distribution not fully reported)	CDI	Presymptomatic infants showed communicative development within the normal range; symptomatic SMA type 1 children showed delayed expressive vocabulary compared with normative data	Early treatment and presymptomatic diagnosis were associated with more typical communicative development trajectories	7
Brusa et al., 2025	37	Range 4–9 years	51%	SMA type 1 (subtypes 1a, 1b, 1c)	Nusinersen, onasemnogene abeparvovec, risdiplam (including treatment switches; distribution not fully reported)	MB-CDIs; SCQ	Most children acquired expressive language, although often below normative levels; SMA1c showed better language performance than SMA1b. Approximately 11% scored above the SCQ cut-off, indicating possible social communication impairments	Children with treated SMA type 1 can develop expressive language, albeit delayed, and a subset shows social communication difficulties, particularly those with more severe language impairment	8
Díaz-López et al., 2025	32	26.7 months (SD NR)	NR	SMA Type I	NR	PEDI-CAT (Social/Cognitive domain)	Gradual improvements in social/cognitive functioning, reaching clinically meaningful change only at weeks 8–16	Early powered mobility training improved functional independence (daily activities and mobility), with modest gains in participation and reductions in parental stress; quality of life changes did not exceed measurement error thresholds	6
Gandolfi et al., 2025	41	Median age at cognitive assessment 32.9 months (range 18.0–118.8)	63.4%	SMA type 1 (subtypes: 1a 41.5%, 1b 39.0%, 1c 19.5%)	Nusinersen (39%), Risdiplam (19.5%), Onasemnogene abeparvovec (41.5%)	Bayley-III, WPPSI-IV, WISC-IV (age-appropriate cognitive tests)	Median DQ/IQ = 85 (range 55–120)	Swallowing function remained stable over 12 months despite significant improvement in motor function (CHOP-INTEND); moderate association between cognitive function and motor and swallowing efficacy scores	4
Guibert et al., 2026	16	Range 2.6–15.4 years (mean not reported)	37.5%	Type I: 5; Type II: 5; Type III: 6	Nusinersen (*n* = 8), Risdiplam (*n* = 3), Gene therapy (onasemnogene abeparvovec) (*n* = 5)	WPPSI-IV, WISC-V, FEE, BRIEF questionnaire, NEPSY-II, CBCL; language assessment with EVALO and EVALEO batteries	Mean IQ 102 ± 4 (no intellectual disability); ~15% executive dysfunction; oral language disorders in 73%; written language disorders in 29%	Intellectual functioning generally preserved, but frequent language impairments and some executive vulnerabilities; language deficits more common in severe phenotypes and lower SMN2 copies	5
Hu et al., 2023	22 SMA patients (20 healthy controls)	NR (adult ≥18 years)	NR	SMA type III	No disease-modifying therapy	MoCA; VFT; WCST; ANT	SMA patients showed lower performance than controls in verbal fluency and executive function tasks (Stroop and WCST). Deficits were also found in executive control networks in ANT.	Adult patients with SMA III showed selective executive function impairment, associated with younger age at onset, worse motor function, and higher depression/anxiety levels. Ambulant patients performed better than non-ambulant patients.	6
Kizina et al., 2021	33	35 ± 11 years	36%	SMA type 1 (*n* = 1), type 2 (*n* = 15), type 3 (*n* = 17)	Nusinersen (all patients)	WAIS-IV	VCI: 96.21 ± 12.43; PRI: 95.61 ± 14.76; WMI: 95.82 ± 15.72 (overall cohort). SMA type 2 showed lower PRI (90.73 ± 12.58) and WMI (90.33 ± 12.95) vs. normative population	Overall IQ domains similar to general population; SMA type 2 showed reduced working memory and perceptual reasoning compared with normative values	8
Kölbel et al., 2024	40	29.25 ± 4.86 months	57.5%	5q-SMA (patients with 2, 3, ≥4 SMN2 copies; detected by newborn screening)	Mainly nusinersen (some switched to onasemnogene abeparvovec; 4 untreated)	BSID-III	Mean scores: cognition 94.55 ± 24.01; language 86.09 ± 26.41; motor 81.28 ± 28.07. 35% below-average cognition (10/14 had 2 SMN2 copies)	Cognitive development associated with SMN2 copy number; children with 2 SMN2 copies more likely to have below-average cognitive scores despite early therapy	8
Lenzoni et al., 2022	22 SMA patients (plus 22 healthy controls)	Median age 29 (IQR 22–39)	50%	SMA type III (IIIa *n* = 7; IIIb *n* = 15)	Not reported (patients untreated in study context)	Comprehensive neuropsychological battery: Raven Progressive Matrices, Phonemic Fluency, Digit Span (forward/backward), Stroop Test, Trail Making Test A/B, Boston Naming Test, Semantic Fluency, Rey–Osterrieth Complex Figure Test, Rey Auditory Verbal Learning Test, MMSE	SMA patients performed worse than controls in visuospatial abilities (ROCFT), executive function (TMT-B), reasoning (RPM), and language (BNT). Language domain z-scores were significantly lower.	Cognitive performance in SMA III is associated with clinical factors and motor severity. Patients with greater motor impairment showed lower attention but relatively higher language, verbal fluency, and memory performance, suggesting compensatory mechanisms and possible CNS involvement.	6
Mix et al., 2021	31	Median 35.9 years (range 19.1–64.6)	35.5%	SMA type II-III	Majority treated with nusinersen	ECAS; RMET; additional language and executive tests	No significant cognitive impairment compared with controls; cognitive performance overall preserved	Executive function was inversely correlated with motor impairment (patients with worse motor function showed better executive performance, possibly reflecting compensatory cognitive mechanisms)	7
Ngawa et al., 2023	18 (11 SMA1 symptomatic + 7 presymptomatic SMA)	Not clearly reported; infants and toddlers (assessed longitudinally from infancy)	NR	SMA type 1 (symptomatic) and presymptomatic SMA	Mainly nusinersen, some patients later switched to risdiplam	Bayley-III	Pre-symptomatically treated patients mostly scored within the average cognitive and communicative ranges. Among post-symptomatically treated SMA1 patients, several had low-average or abnormal scores, particularly in communication and cognitive domains.	Early (presymptomatic) treatment is associated with better developmental trajectories, while patients treated after symptom onset show more variable and sometimes reduced cognitive/communication scores. Early treatment may preserve neurodevelopmental outcomes.	7
Osmanovic et al., 2020	34	40.2 ± 12.2 years	32.4%	SMA type 2–4	Some patients treated with nusinersen (antisense therapy)	ECAS; WST	SMA patients showed higher ECAS total scores and better performance in memory and ALS-specific domains compared with ALS patients	Adult SMA patients showed overall preserved or superior cognitive performance compared with ALS patients; higher education level may contribute to cognitive advantage	8
Polido et al., 2017	12 SMA-I (24 total incl. controls)	6.0 ± 2.3 years	25%	SMA type I	None reported (pre–disease-modifying therapy era)	Eye-tracker–based pair-matching cognitive tasks (objects, colors, letters, numbers); clinical scales: CHOP INTEND, PEDI, PedsQL	SMA-I children showed significantly lower percentage of correct answers and longer completion times compared with controls in pair-matching tasks	Children with SMA-I demonstrated poorer performance in visual-cognitive matching tasks, and performance correlated with PEDI social function, suggesting reduced environmental interaction may contribute to cognitive difficulties	7
Steffens et al., 2024	20	Mean 37.8 months (median 36; range 24–60)	60%	SMA type 1 with 2-3 SMN2 copies (19 symptomatic, 1 presymptomatic)	Nusinersen, risdiplam, or gene therapy (distribution not fully reported)	BSID-III; WPPSI-IV	11/20 (55%) showed subnormal cognition (<85); 9 had moderate–severe impairment (≤70); 3 had above-average scores (>115)	Cognitive outcomes were heterogeneous in treated SMA type 1 patients; risk factors for poorer cognition included male sex, need for assisted ventilation, and feeding support	5
Tosi et al., 2023	15	2.6 years (range 0.47–6.78)	53.3%	SMA type I (Ia, Ib, Ic)	Mostly Nusinersen (80%); also gene therapy (onasemnogene abeparvovec) and Risdiplam	Griffiths III developmental scales; vineland adaptive behavior scales-II	Mean developmental quotient 64.8; 60% < 70 indicating developmental delay. Lowest scores in gross motor domain; relatively preserved language and learning abilities. Adaptive functioning mean 68.67 (global delay).	Most children show global developmental delay mainly driven by motor impairment, while language and cognitive learning domains show relatively preserved or positive trajectories. Neurocognitive evaluation should be routinely performed in SMA type I patients treated with DMT.	6
Vidovic et al., 2023	23	38.1 ± 11.4 years	43.5%	SMA type 2 (*n* = 9), type 3 (*n* = 14)	Nusinersen	ECAS	Most patients showed cognitive performance within the normal range. Some impairments were observed in ALS-specific domains (especially verbal fluency and executive functions). After 14 months of treatment, ECAS scores improved significantly in several domains, but these changes were interpreted as likely practice effects rather than clinically meaningful improvement.	Adult SMA patients may show mild abnormalities in ALS-specific cognitive domains, but overall cognition is largely preserved. Nusinersen treatment did not produce clinically significant cognitive changes and did not worsen cognition.	7
von Gontard et al., 2002	96	11.2 ± 4.7 years	51%	SMA type I–III	None (pre-DMT era)	K-ABC	Mean IQ (Raven): 109.6 ± 17.0; K-ABC mental processing composite: 103.8 ± 12.6; verbal IQ (Wechsler): 113.8 ± 13.4	Children with SMA show normal general intelligence; adolescents display higher verbal IQ compared with controls, suggesting compensatory development of verbal and cognitive skills	7
Zappa et al., 2021	22	Median 5 years (range 3–11)	64%	SMA type 1 (subtypes: 1a 9%, 1b 36%, 1c 55%)	None (natural history cohort; data collected before SMN-restoring treatments)	RCPM for IQ; TCGB for morphosyntactic comprehension	Median IQ 120 (range 100–130); morphosyntactic comprehension generally in normal range	Cognitive abilities and language comprehension preserved in SMA1 children despite severe motor impairment; speech impairment correlated with motor severity (CHOP-INTEND)	6

NOS scores range from 0 to 8; higher scores indicate better methodological quality. SDQ, strengths and difficulties questionnaire; PedsQL-NM, pediatric quality of life inventory neuromuscular module; ASQ-3, ages and stages questionnaire, third edition; M-CHAT-R, modified checklist for autism in toddlers revised; CDI, MacArthur-Bates communicative development inventories; MB-CDIs, MacArthur-Bates communicative development inventories; SCQ, social communication questionnaire; MoCA, Montreal cognitive assessment; VFT, verbal fluency test; WCST, Wisconsin card sorting test; ANT, attention network test; WAIS-IV, verbal comprehension index, perceptual reasoning index; BSID-III, Bayley scales of infant and toddler development-III; WPPSI-IV, Wechsler preschool and primary scale of intelligence IV; ECAS, Edinburgh cognitive and behavioral ALS screen; K-ABC, raven CPM/SPM, Kaufman assessment battery for children; RCPM, raven colored progressive matrices; TCGB, test di comprensione grammaticale per bambini.

### Global cognitive functioning

Across studies evaluating standardized intellectual measures, overall cognitive functioning was generally preserved in individuals with SMA, particularly in type II and III phenotypes (von Gontard et al., 2002; Buchignani et al., 2023; Kizina et al., 2021). Mean full-scale IQ scores were typically within the normative range, and several cohorts demonstrated cognitive performance comparable to or slightly higher than that of reference populations.

However, index-level analyses reveal a more nuanced picture. Studies using the Wechsler scales consistently identify a within-profile dissociation: Verbal Comprehension Index scores tend to remain within or near normative ranges, whereas Working Memory and Processing Speed indices show selective reductions, particularly in SMA type II. Kizina et al. (2021), using the WAIS-IV in an adult cohort, reported mean Verbal Comprehension Index scores within normal limits, while the Perceptual Reasoning Index and Working Memory Index were significantly lower than normative values in the SMA type II subgroup. Buchignani et al. (2023), using the WISC-IV in school-age children with SMA types II and III, similarly identified Working Memory and Processing Speed as the most vulnerable indices, with a significant correlation between Processing Speed and the degree of motor impairment. In SMA type I, subscale data from the Griffiths III Scales of Child Development reveal a parallel dissociation: the Gross Motor subscale consistently yields the lowest scores and is the primary driver of overall developmental delay, while the Language and Communication and Foundations of Learning subscales follow comparatively more favorable trajectories. Tosi et al. (2023) reported that despite a mean developmental quotient of 64.8, the Language and Learning domains showed relatively preserved or positive trends. Bitetti et al. (2024) documented significant post-treatment gains in the Language and Communication, Foundations of Learning, Eye-Hand Coordination, and Personal-Social–Emotional subscales, with no parallel improvement in the Gross Motor domain, underscoring that global developmental delay in SMA type I is largely motor-driven rather than a reflection of uniform cognitive impairment.

However, a minority of patients presented borderline intellectual functioning or mild impairment, more frequently in association with severe motor disability or early disease onset (Buchignani et al., 2025; Steffens et al., 2024). Variability in results partly reflected methodological differences in assessment tools and the need for task adaptation in patients with significant motor or speech limitations.

### Executive functions and processing domains

Selective cognitive vulnerabilities were consistently reported in executive domains, including reduced processing speed, working memory inefficiencies, and difficulties in attentional control (Mix et al., 2021; Lenzoni et al., 2022; Hu et al., 2023).

Reduced processing speed, working memory inefficiencies, and difficulties in attentional control emerged as recurrent findings across pediatric and adult samples.

These alterations were often subtle and did not necessarily translate into global intellectual deficits but were associated with functional challenges in academic and everyday settings.

Notably, these executive and processing vulnerabilities are detectable not only through dedicated neuropsychological batteries but also within standardized intelligence scales: the selective reduction of Working Memory and Processing Speed indices in the context of preserved Verbal Comprehension—documented across both pediatric and adult Wechsler-based studies—aligns with findings from batteries specifically targeting executive functions (Lenzoni et al., 2022; Mix et al., 2021; Hu et al., 2023), reinforcing the consistency of this cognitive pattern across assessment approaches.

Studies employing comprehensive neuropsychological batteries highlighted impairments in verbal fluency, cognitive flexibility, and sustained attention, suggesting potential involvement of fronto-subcortical networks (Lenzoni et al., 2022; Hu et al., 2023).

### Language and learning abilities

Language development represented one of the most frequently affected domains (Buchignani et al., 2024; Brusa et al., 2025; Guibert et al., 2026). Several pediatric studies documented delays in expressive vocabulary, oral language production, or written language acquisition despite preserved global cognitive performance.

Learning difficulties were particularly evident in patients with severe phenotypes or limited motor autonomy, potentially reflecting both neurodevelopmental factors and environmental constraints such as reduced participation in typical school activities (Guibert et al., 2026; Buchignani et al., 2025). In contrast, patients with milder phenotypes often achieved age-appropriate educational milestones, although requiring adaptive strategies or individualized support.

### Behavioral and socio-emotional functioning

Behavioral and emotional difficulties were variably described across studies. Increased rates of hyperactivity, anxiety symptoms, social withdrawal, and adaptive challenges were reported in pediatric cohorts (Balaji et al., 2026; Buchignani et al., 2025).

In adult populations, subtle executive and socio-emotional alterations were occasionally observed, including reduced social participation and cognitive fatigue. These findings suggest that neuropsychological functioning in SMA extends beyond purely cognitive domains and may involve broader psychosocial adaptation processes.

### Neurodevelopmental outcomes in early-onset SMA

Studies focusing on SMA type I in the treatment era reported highly heterogeneous developmental trajectories (Ngawa et al., 2023; Steffens et al., 2024; Tosi et al., 2023). While some children exhibited global developmental delay, others demonstrated relatively preserved communicative and cognitive skills, particularly when treated pre-symptomatically or early after symptom onset (Ngawa et al., 2023; Buchignani et al., 2024).

This variability underscores the emerging complexity of neurodevelopmental phenotypes in SMA and highlights the importance of longitudinal monitoring as survival and motor function improve.

### Methodological heterogeneity

Considerable heterogeneity was observed in study design, cognitive assessment tools, and patient characteristics. Motor impairment, fatigue, communication limitations, and differences in testing adaptations were frequently identified as potential confounders influencing neuropsychological outcomes.

Across phenotype categories, language and executive function batteries are the domains most consistently assessed beyond global IQ measures. SMA type I studies rely predominantly on developmental scales sensitive to motor confounding (Griffiths III, Bayley-III), while type II-III studies use Wechsler-based intelligence tests, limiting direct cross-phenotype comparison. Importantly, the cognitive patterns identified across these diverse instruments converge on common vulnerabilities, suggesting that the dissociation between preserved verbal abilities and reduced processing/working memory reflects a genuine neurobiological signal rather than a methodological artifact.

## Discussion

This scoping review provides an updated synthesis of current evidence on cognitive and neurodevelopmental outcomes in spinal muscular atrophy (SMA), highlighting the complexity and heterogeneity of neuropsychological trajectories across phenotypes and therapeutic eras.

Across the 23 included studies, three cross-cutting patterns emerge. First, a within-profile cognitive dissociation—preserved verbal comprehension alongside selective reductions in working memory, processing speed, and executive functions—is documented consistently across SMA types II and III, in both pediatric and adult samples, and across heterogeneous assessment batteries. Second, neurodevelopmental heterogeneity increases substantially with phenotypic severity: outcomes in types II and III are relatively predictable, whereas early-onset type I shows a wide spectrum from near-typical development to global delay. Third, treatment timing exerts a measurable influence: pre-symptomatic and early-treated patients demonstrate systematically more favorable trajectories than those treated after symptom onset.

Traditionally conceptualized as a motor neuron disorder, SMA is increasingly recognized as a multisystem condition with potential central nervous system involvement (Detering et al., 2022). The present findings support this evolving perspective, suggesting that cognitive and behavioral functioning represent relevant clinical domains requiring systematic evaluation, particularly as disease-modifying therapies modify survival and functional outcomes.

Consistent with earlier literature, global intellectual functioning appears largely preserved in individuals with SMA type II and III (von Gontard et al., 2002; Mix et al., 2021; Lenzoni et al., 2022).

Across several studies included in this review, mean full-scale IQ scores fell within normative ranges; however, index-level analyses consistently revealed a within-profile dissociation, with verbal comprehension and crystallized abilities remaining relatively intact while working memory, processing speed, and perceptual reasoning showed selective vulnerability, particularly in SMA type II. This pattern reinforces the concept that motor disability does not necessarily imply global cognitive impairment, but also cautions against interpreting preserved full-scale IQ as indicative of a uniformly intact cognitive profile.

These within-profile vulnerabilities, while not necessarily impairing global intellectual performance, carry practical implications for academic functioning, adaptive autonomy, and daily participation. Such findings are consistent with neurobiological hypotheses suggesting that SMN protein deficiency may influence broader neural networks beyond anterior horn motor neurons. Moreover, environmental factors related to physical disability—including reduced exploration, limited peer interaction, and increased dependency—may contribute to developmental variability. The interaction between biological and contextual determinants likely explains the heterogeneous cognitive phenotypes observed across studies.

It is noteworthy that executive vulnerabilities are documented both in untreated adult cohorts with type III (Lenzoni et al., 2022; Hu et al., 2023) and in pediatric cohorts with type II-III under nusinersen treatment (Buchignani et al., 2023; Mix et al., 2021), suggesting that these deficits are not solely attributable to disease chronicity or the absence of treatment, but may reflect an underlying neurobiological substrate independent of motor neuron preservation.

Interpreting cognitive findings in SMA requires distinguishing among at least three levels of explanation. The first is true CNS involvement: SMN protein deficiency disrupts processes—including hippocampal neurogenesis, snRNP biogenesis, and synaptic mRNA translation—that are relevant to brain development independently of motor neuron loss (Detering et al., 2022; Wishart et al., 2010). Executive and language deficits that are documented in patients with preserved or near-normal global IQ, and that correlate with neuroimaging findings in subcortical structures (Lécuyer et al., 2023), are consistent with this level of explanation. The second level is motor-related assessment bias: standard cognitive instruments frequently require timed motor responses, graphomotor execution, or oral production, all of which are systematically limited in SMA patients, particularly those with type I or severe type II phenotypes. This bias can lead both to underestimation of genuine cognitive abilities and to apparent deficits that disappear when motor-adapted tools are used (Giannotta et al., 2024). The third level encompasses environmental and contextual factors: reduced physical exploration, limited peer interaction, frequent hospitalizations, and increased caregiver dependency may all modulate developmental trajectories, particularly in early-onset disease. Distinguishing among these levels is not merely a methodological issue—it has direct clinical implications, since deficits arising from each level call for different interventions.

Preclinical evidence further supports CNS involvement beyond the motor axis. In SMA mouse models, impaired hippocampal neurogenesis and widespread proteomic dysregulation have been documented when SMN levels are reduced (Wishart et al., 2010), implicating cortico-hippocampal networks in the neurodevelopmental phenotype. More recently, cerebellar pathology has been identified as an additional substrate: cerebellar abnormalities contribute to neurodevelopmental, social, and communication deficits in SMA mice independently of spinal motor neuron involvement, with these alterations being largely restricted to severe SMA models (Gerstner et al., 2026).

This preclinical convergence aligns with the clinical observation that neurodevelopmental heterogeneity and severity are substantially greater in type I than in milder phenotypes, and suggests that cortico-hippocampal and cerebellar circuits represent priority targets for neurobiological investigation in treated SMA populations.

In contrast, neurodevelopmental outcomes in early-onset SMA, particularly type I, appear considerably more variable. The introduction of disease-modifying therapies has profoundly changed the natural history of the disease (Mercuri et al., 2022; Pera and Mercuri, 2024), enabling longitudinal observation of developmental trajectories in children who previously would not have survived beyond infancy. Within this emerging clinical scenario, some treated patients demonstrate relatively preserved cognitive and communicative abilities, while others present global developmental delays or specific language and learning difficulties.

This variability likely reflects multiple interacting factors, including treatment timing, disease severity, SMN2 copy number, environmental stimulation, and access to early rehabilitation. Importantly, pre-symptomatic or early-treated children appear to show more favorable developmental trajectories, highlighting the critical importance of newborn screening and early therapeutic intervention.

In contrast to the relatively predictable cognitive profiles observed in type II and III—where the within-profile dissociation between verbal and processing/working memory abilities is a consistent finding across untreated, recently treated, and long-term treated adults—type I outcomes in the treatment era show a genuinely wide spectrum. This heterogeneity is partly explained by treatment timing: studies comparing pre-symptomatic versus post-symptomatic cohorts (Ngawa et al., 2023; Buchignani et al., 2024; Kölbel et al., 2024) consistently demonstrate more favorable communicative and cognitive trajectories in children treated before symptom onset, underscoring that the window of CNS vulnerability may be time-sensitive and not fully addressed by late therapeutic intervention. Language and communication domains emerged as particularly relevant areas of vulnerability.

A specific dimension warranting closer attention is socio-communicative functioning and the possible co-occurrence of autism spectrum disorder (ASD). Several studies have begun to investigate this domain systematically. Buchignani et al. (2025) identified ASD in approximately 10% of children with SMA type I and in those with pre-symptomatic SMA, using the SCQ, CARS2, and DSM-5 criteria—a prevalence slightly exceeding general population rates. Brusa et al. (2025) found that approximately 11% of treated SMA type I children scored above the SCQ cut-off for possible social-communication impairment, with greater difficulties observed in those with more severe expressive language deficits. Coratti et al. (2025) and Baranello and Neurodevelopment in SMA Working Group (2024) have further highlighted ASD and broader neurodevelopmental comorbidities as an emerging clinical concern in the contemporary treatment era.

Nevertheless, the available evidence does not yet support the characterization of a defined ASD trajectory in SMA. The studies differ substantially in the instruments used, the diagnostic thresholds applied, and the extent to which motor and speech impairments may have confounded socio-communicative assessment. Longitudinal data are lacking, and it remains unclear whether the observed difficulties reflect true ASD comorbidity, a distinct neurodevelopmental phenotype related to SMN deficiency and its effects on brain maturation, or are partially an artifact of assessment bias.

A recently published commentary specifically addressing this challenge highlights that screening questionnaires used in isolation tend to overestimate the frequency and severity of socio-communicative difficulties in SMA type 1, and that the most accurate diagnostic approach combines questionnaires, structured clinical observation, and formal diagnostic tools applied by a multidisciplinary team (Buchignani and Mercuri, 2026). The same authors emphasize the need for motor-free assessment instruments, including eye-tracking paradigms and adaptive questionnaires without motor-dependent items, to disentangle true socio-communicative impairment from the confounding effects of motor and bulbar dysfunction. Future longitudinal studies should incorporate such adapted tools to clarify whether the observed socio-communicative difficulties represent genuine ASD comorbidity, a distinct neurodevelopmental phenotype related to SMN deficiency, or partly an artifact of motor-biased assessment.

Several studies reported expressive language delays, oral-motor speech difficulties, or written language impairments, even in the context of preserved global intellectual functioning. These observations align with recent empirical reports suggesting increased prevalence of neurodevelopmental and social communication challenges in treated SMA populations (Coratti et al., 2025), as well as with expert consensus highlighting the clinical relevance of these emerging comorbidities (Baranello and Neurodevelopment in SMA Working Group, 2024). Such findings underscore the need for integrated neurodevelopmental monitoring within multidisciplinary SMA care pathways.

Behavioral and emotional adaptation also represents a clinically significant dimension. Increased rates of anxiety, attentional difficulties, and social participation challenges were described in pediatric cohorts. While these difficulties may partly reflect disease-related stress and physical limitations, they may also be influenced by environmental factors, educational barriers, and evolving psychosocial expectations as survival improves.

Importantly, the interpretation of cognitive outcomes in SMA must consider the profound transformation introduced by disease-modifying therapies. Historically, cognitive research in SMA was largely limited to observational data from patients with milder phenotypes, due to the high mortality associated with severe forms. In the contemporary therapeutic era, improved survival has expanded the clinical spectrum and revealed previously unrecognized developmental variability.

Methodological heterogeneity across studies represents an additional key issue. Differences in sample size, assessment tools, testing adaptations, and inclusion criteria complicate direct comparison of findings. Motor limitations, fatigue, and communication difficulties may introduce significant bias in cognitive testing, potentially leading to under- or overestimation of specific abilities. These limitations emphasize the importance of developing standardized neuropsychological protocols specifically tailored to neuromuscular populations (Giannotta et al., 2024).

From a clinical perspective, the present findings suggest that cognitive monitoring should become an integral component of SMA management, particularly in pediatric populations. Early identification of language or executive difficulties may facilitate targeted educational and rehabilitation interventions, ultimately supporting long-term functional autonomy and psychosocial well-being. Beyond routine monitoring, intellectual development and communicative functioning should be explicitly considered as clinically meaningful outcomes in treated SMA type I populations: given the greater vulnerability observed in early-onset disease, these domains should not be regarded as secondary consequences of motor impairment, but rather as integral components of the disease phenotype in the therapeutic era. Future studies should therefore specifically investigate whether disease-modifying therapies can influence neurodevelopmental trajectories—including cognition, language, and adaptive functioning—positioning these domains as potential efficacy outcomes alongside motor and survival endpoints. Structured cognitive and communicative assessments, together with early parental guidance and environmental stimulation, should be incorporated into standard of care.

## Conclusion

Current evidence indicates that global intellectual functioning is largely preserved in milder SMA phenotypes (types II and III), but this should not be interpreted as indicative of a uniformly intact cognitive profile. Domain-specific vulnerabilities—particularly in processing speed, working memory, executive functions, and language—are documented consistently across phenotypes, age groups, and assessment approaches, and carry practical implications for academic functioning and daily participation. Neurodevelopmental outcomes in early-onset SMA remain substantially heterogeneous in the treatment era, with a meaningful proportion of treated type I patients showing language delay, executive difficulties, or global developmental delay.

As survival improves and treated children reach school age and adolescence, integrating neurodevelopmental monitoring into routine SMA care will be essential to optimize long-term functional and psychosocial outcomes. Future research should focus on longitudinal cohort studies integrating neuropsychological assessment, neuroimaging biomarkers, treatment timing, and environmental variables. Such approaches will be essential to clarify the mechanisms underlying cognitive variability and to optimize individualized care strategies in the evolving therapeutic landscape of SMA.
